# Brucellosis Vaccines: Assessment of *Brucella melitensis* Lipopolysaccharide Rough Mutants Defective in Core and O-Polysaccharide Synthesis and Export

**DOI:** 10.1371/journal.pone.0002760

**Published:** 2008-07-23

**Authors:** David González, María-Jesús Grilló, María-Jesús De Miguel, Tara Ali, Vilma Arce-Gorvel, Rose-May Delrue, Raquel Conde-Álvarez, Pilar Muñoz, Ignacio López-Goñi, Maite Iriarte, Clara-M. Marín, Andrej Weintraub, Göran Widmalm, Michel Zygmunt, Jean-Jacques Letesson, Jean-Pierre Gorvel, José-María Blasco, Ignacio Moriyón

**Affiliations:** 1 Department of Microbiology and Parasitology, University of Navarra, Pamplona, Spain; 2 Instituto de Agrobiotecnología, CSIC-UPNA-Gobierno de Navarra, Pamplona, Spain; 3 Centro de Investigación y Tecnología Agroalimentaria (CITA), Sanidad Animal, Gobierno de Aragón, Zaragoza, Spain; 4 Arrhenius Laboratory, Stockholm University, Stockholm, Sweden; 5 Centre d'Immunologie de Marseille-Luminy, Aix Marseille Université, Faculté de Sciences de Luminy, Marseille, France; 6 INSERM, U631, Marseille, France; 7 CNRS, UMR6102, Marseille, France; 8 Laboratoire d'Immunologie et Microbiologie - Unité de Recherche en Biologie Moléculaire (URBM), Facultés Universitaires - Notre-Dame de la Paix (FUNDP), Namur, Belgium; 9 Karolinska Institute, Department Laboratory Medicine, Division of Clinical Bacteriology, Karolinska University Hospital, Stockholm, Sweden; 10 INRA, UR1282, Infectiologie Animale et Santé Publique, IASP, Nouzilly, France; University of Minnesota, United States of America

## Abstract

**Background:**

The brucellae are facultative intracellular bacteria that cause brucellosis, one of the major neglected zoonoses. In endemic areas, vaccination is the only effective way to control this disease. *Brucella melitensis* Rev 1 is a vaccine effective against the brucellosis of sheep and goat caused by *B. melitensis*, the commonest source of human infection. However, Rev 1 carries a smooth lipopolysaccharide with an O-polysaccharide that elicits antibodies interfering in serodiagnosis, a major problem in eradication campaigns. Because of this, rough *Brucella* mutants lacking the O-polysaccharide have been proposed as vaccines.

**Methodology/Principal Findings:**

To examine the possibilities of rough vaccines, we screened *B. melitensis* for lipopolysaccharide genes and obtained mutants representing all main rough phenotypes with regard to core oligosaccharide and O-polysaccharide synthesis and export. Using the mouse model, mutants were classified into four attenuation patterns according to their multiplication and persistence in spleens at different doses. In macrophages, mutants belonging to three of these attenuation patterns reached the *Brucella* characteristic intracellular niche and multiplied intracellularly, suggesting that they could be suitable vaccine candidates. Virulence patterns, intracellular behavior and lipopolysaccharide defects roughly correlated with the degree of protection afforded by the mutants upon intraperitoneal vaccination of mice. However, when vaccination was applied by the subcutaneous route, only two mutants matched the protection obtained with Rev 1 albeit at doses one thousand fold higher than this reference vaccine. These mutants, which were blocked in O-polysaccharide export and accumulated internal O-polysaccharides, stimulated weak anti-smooth lipopolysaccharide antibodies.

**Conclusions/Significance:**

The results demonstrate that no rough mutant is equal to Rev 1 in laboratory models and question the notion that rough vaccines are suitable for the control of brucellosis in endemic areas.

## Introduction

Brucellosis is a group of closely related zoonotic bacterial diseases caused by the members of the genus *Brucella*, a group of gram-negative bacteria that behave as facultative intracellular parasites. There are several *Brucella* species, and they infect a wide range of mammals in which they are a main cause of abortions and infertility. In addition, they are readily transmitted to human beings where they produce a grave and debilitating disease that requires a long and costly antibiotic therapy and that often leaves permanent sequelae [1]. Because of its high incidence in developing countries, economic consequences, and difficult eradication, the World Health Organization considers brucellosis as one of the seven neglected zoonoses, a group of diseases that contribute to the perpetuation of poverty [2].

Ruminants are highly susceptible to brucellosis. Cattle are most often infected by *B. abortus* whereas sheep and goats are the preferred hosts of *B. melitensis*, the *Brucella* species most virulent for humans [3]. Although there is no human vaccine, vaccination of animals against brucellosis is one of the most cost-effective measures to improve human health in endemic areas [4] as well as an essential tool to achieve eradication [5], [6]. For these purposes, vaccines *B. abortus* S19 and *B. melitensis* Rev 1 have been successfully used in some developed countries, but both induce abortions when applied during pregnancy, are virulent for humans and elicit antibodies to the smooth (S) lipopolysaccharide (LPS) of the *Brucella* surface that interfere in serodiagnosis. Moreover, Rev 1 is resistant to streptomycin, an antibiotic used to treat the disease. Although the serodiagnosis problem can be partially solved by using the conjunctival route, by avoiding adult vaccination, and by an individual serological follow up, the breeding conditions characteristic of small ruminants make these measures unrealistic in large areas of the world. Therefore, effective brucellosis vaccines not interfering in diagnosis would represent a major breakthrough [7], [8].

Rough (R) *Brucella* mutants lack the LPS immunodominant N-formylperosamine O-polysaccharide (O-PS) and are attenuated. Thus, they have been the subject of great attention as alternative vaccines [7], [8]. Some R vaccines or candidates are spontaneous mutants selected after repeated passage on antibiotic-containing media. This approach was used to obtain RB51, a *B. abortus* R mutant that carries a IS711-disrupted *wboA* (putative glycosyltranferase gene) as well as unknown mutations also affecting LPS. However, RB51 has yielded controversial results in cattle, is not effective in sheep and is resistant to rifampin, an antibiotic used to treat brucellosis [8]. *B. melitensis* RBM9, RBM11, RBM15, RBM17 and RBM19 have been obtained by a similar method and, as expected, they carry undefined LPS defects and are rifampin resistant [9]. Targeted and transposon mutagenesis have also been used. Disruption of *per*, *wboA* and *wbkA* (putative perosamine synthetase and glycosyltranferase genes) results in R mutants that outperform RB51 in the mouse model [10]–[12], showing that empirically R vaccines can be improved. However, R mutants can result from mutations affecting O-PS precursor synthesis, its polymerization and transport or from a variety of defects in the inner core oligosaccharide. Presently, it is not known which of these mutations results in the best R vaccine or how such a vaccine would compare with S19 or Rev 1. To address these questions, we investigated 14 LPS *B. melitensis* mutants with respect to LPS defects, intracellular multiplication and virulence and vaccine efficacy in mice. The results provide the basis for selecting the best R candidates for sheep and goat vaccination against *B. melitensis*, and are also relevant for an appraisal of the value of brucellosis R vaccines.

## Results

### Mutagenesis, mutant selection and growth characteristics

Perusal of the literature shows that 16M (reference strain of *B. melitensis* biovar 1 and the one sequenced) does not always show the virulence levels of other S *brucellae* [12]–[14]. Because of this, we used this strain and also *B. melitensis* H38 (fully virulent [15]) to obtain the nalidixic acid resistant (Nal^R^) derivatives necessary for mutagenesis and confirmed that this selection did not affect their characteristics in mice (not shown). Then, we performed transposon mutagenesis and obtained ca. 16,500 kanamycin resistant mutants from which we selected 23 that displayed a stable R phenotype both *in vitro* and in mice, as judged by the crystal violet test, lack of reactivity with anti O-PS antibodies and phage typing. These mutants mapped in 16 open reading frames (ORFs) scattered into eight regions. Complementation failed to restore the S phenotype in mutants in four of these ORFs. Moreover, mutagenesis of the adjacent ORFs ruled out both possible polarity effects and the involvement in LPS synthesis of some ORFs with suggestive annotations (Table S1). We confirmed the R phenotype of the remaining mutants (Table 1) by examining the LPS by the sodium dodecylsulfate-proteinase K method and, for ORFs BMEI1326, BMEI1414, BMEI1426 and BMEI1427, by making in frame deletion mutants. Finally, since some mutants showed differences in colony size suggestive of altered growth rates, we obtained the corresponding growth curves. Mutants in BMEI1415 (*wzm*), BMEI1886 (p*gm*) and BMEI1326 (wa**) were retarded with respect to the parental strain whereas BMEII0899 (*manB_core_*) grew faster (Figure S1).

**Table 1:** ORFs shown to be involved in *B. melitensis* LPS synthesis.

ORF	Strain (n° mutants) 1	Mutagenesis	Role (R phenotype)	LPS gene	Annotation
BMEI0997	16M (1)	Transposon	O-PS synthesis (R1)	*wboB*	Mannosyltransferase
BMEI0998	H38 (1)	Transposon	O-PS synthesis (R1)	*wboA*	Mannosyltransferase
BMEI1326	16M (1)	Transposon and in frame deletion	Core synthesis (R2)	wa**	Glycosyltransferase
BMEI1393	H38 (2),16M (1)	Transposon	O-PS synthesis (R1)	*wbkE*	Mannosyltransferase
BMEI1396	16M (1)	Targeted mutagenesis	Uncertain	*manB*	Phosphomannomutase
BMEI1404	16M (1)	Transposon	O-PS synthesis (R1)	*wbkA*	Mannosyltransferase
BMEI1413	16M (1)	Transposon	O-PS synthesis (R1)	*gmd*	GDP-mannose dehydratase
BMEI1414	16M (1), H38 (2)	Transposon and in frame deletion	O-PS synthesis (R1)	*per*	Perosamine synthetase
BMEI1415	16M (1)	Transposon	O-PS synthesis (R1)	*wzm*	ABC transporter
BMEI1426	H38 (3)	Transposon and in frame deletion	O-PS synthesis (R1)	*wbkF*	Undecaprenyl-glycosyltransferase
BMEI1427	H38 (2)	Transposon and in frame deletion	O-PS synthesis (R1)	*wbkD*	Epimerase/dehydratase
BMEI1886	16M (1)	Transposon	Core synthesis (R2)	*pgm*	Phosphoglucomutase
BMEII0899	H38 (1)	Transposon	Core synthesis (R3)	*manB*_core_	Phosphomannomutase

1 16M, *B. melitensis* 16M Nal^R^; H38, *B. melitensis* H38Nal^R^

2 n.a., not applicable.

### The mutations block the major LPS polysaccharide synthesis pathways

**Figure 1:**
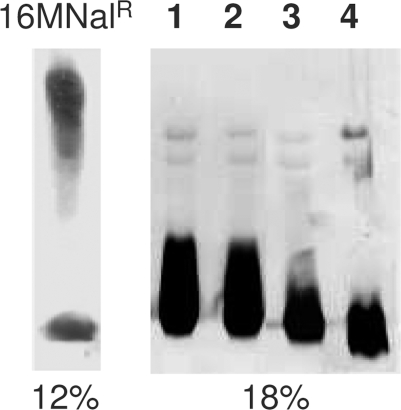
LPS profiles by SDS-PAGE. SDS-proteinase K extracts of the 16M Nal^R^ strain and of mutants representative of the R1 (Bm16MR*wboB* [lane 1] and Bm16MR*per* [lane 2]), R2 (Bm16MR*pgm* [lane 3]) and R3 (BmH38R*manB_core_* [lane 4]) LPS types were analyzed in gels of the indicated acrylamide % and then periodate-silver stained.

In order to assign the mutations to the O-PS or the core oligosaccharide biosynthetic pathways, we first classified the mutants as R1, R2 or R3 according to the decrease in LPS molecular weight (Figure 1 and Table 1). Three R1 mutants mapped in *per* (BMEI1414, Table 1). Since *per* is involved in the synthesis of perosamine [16], the only *Brucella* O-PS sugar [17], we took this as evidence for a complete core in R1 and, consequently, for a mutation affecting the O-PS. Likewise, the progressively defective core in R2 and R3 mutants indicated that the corresponding ORF belonged to core synthesis pathways. Consistent with these interpretations, the 2-keto-3-deoxyoctulosonic acid ([Kdo] inner core marker) content of solvent-extracted R-LPS of BMEI1414 and BMEI1427 (R1), BMEI1326 (R2) and BMEII0899 (R3) mutants was 3.8, 4.0, 6.8 and 9.5%, respectively.

#### Mutants affected in O-PS synthesis

**Figure 2:**
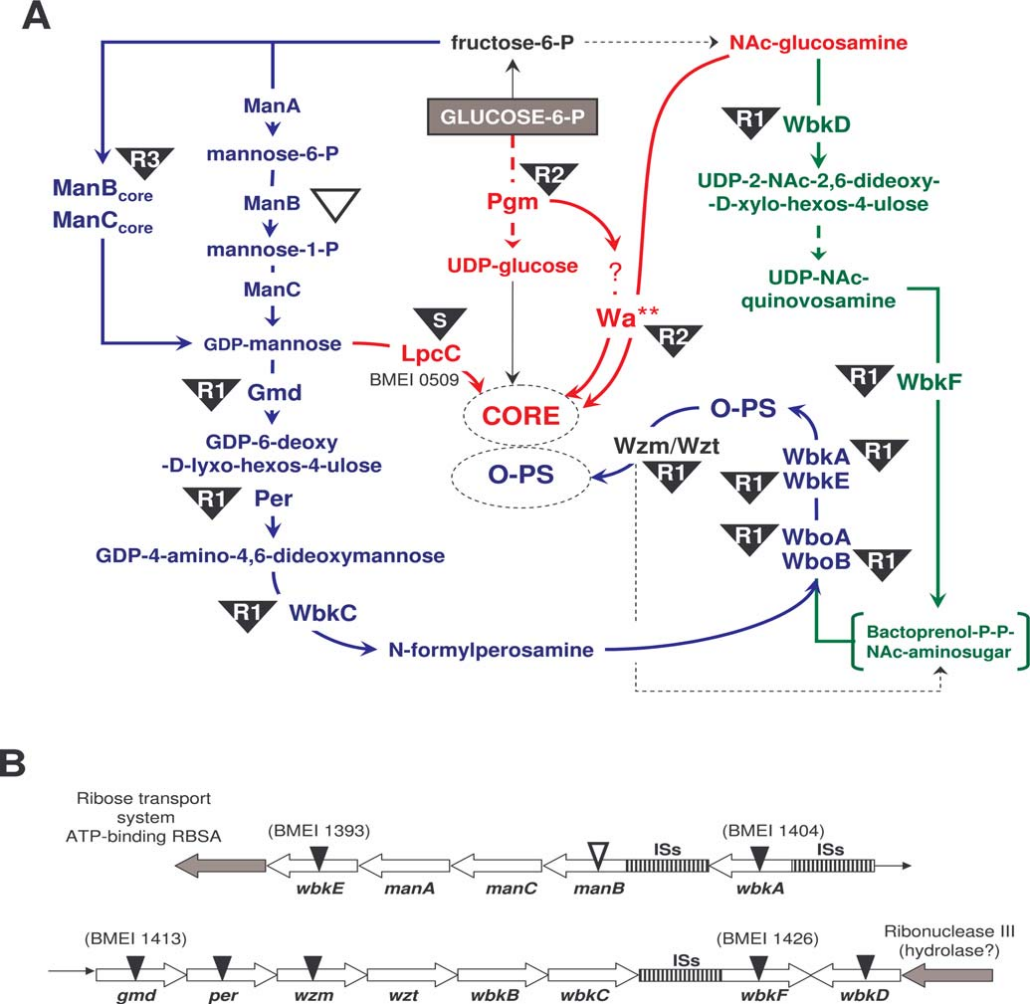
Genetics of *B. melitensis* S-LPS biosynthesis. (A), Pathways. *Brucella* grows with glucose as the only C source and is thus able to derive all S-LPS precursors from this sugar. The steps leading to N-formylperosamine synthesis and to its polymerization by Wbo and Wbk glycosyltransferases are in blue, and those leading to bactroprenol priming for N-formylperosamine polymerization in green. Once this happens, O-PS is translocated to the periplasm by the Wzm/Wzt ABC transporter (also in blue) and ligated to the core oligosaccharide which results from the pathways marked in red. The steps disrupted in this work are indicated by black triangles in which R1, R2, or R3 refer to the LPS phenotypes (the *wbkC* mutant is described in reference [16]). An empty triangle indicates a mutation that does not generate R phenotype, and a black triangle marked with S a mutation that, while blocking the synthesis of a core lateral branch, does not prevent O-PS linkage to the core (Conde-Álvarez, R., unpublished results). (B), The major (*wbk*) genetic region of *Brucella* O-PS synthesis. This region contains genes coding for enzymes necessary for N-formylperosamine synthesis (*gmd*, *per*, *wbkC*), two O-PS glycosyltransferases (*wbkE*, *wbkA*), the ABC transporters (*wzm*, *wzt*), the PNPT enzyme (*wbkD*) and at least one enzyme necessary for the synthesis of an N-acetylaminosugar (*wbkF*), as well as groups of insertion sequences (ISs) that make it unstable. The mutations analyzed in this work are marked with triangles. Mutations in *manB* and *wbkB* do not generate R mutants (this work and reference [16]).

Mutants in BMEI0997 and BMEI0998 and in ORFs from BMEI1393 to BMEI1427 were of the R1 type (Table 1 and Figure 2A). The two first ORFs, putatively encoding glycosyltransferases, included the previously described *wboA* (BMEI0998) [18]. According to their R1 phenotype, these two ORFs are implicated in O-PS synthesis and BMEI0997 was thus named *wboB*. Likewise, the R1 phenotype of BMEI1393 matched its annotation as a mannosyltransferase gene (mannose and perosamine are related) and its location close to *wbkA* (BMEI1404), the first ORF of the O-PS *wbk* region [16] (Figure 2B). Because of this, we named the gene *wbkE*. The R1 phenotype of mutants in genes *wbkA*, *gmd*, *per* and *wzm*, all in the *wbk* region, was also in keeping with the roles proposed in previous works (Table 1 and Figure 2A) [11], [16]. Two other R1 mutants mapped close to *wbk* (Figure 2B). The first one (BMEI1426), separated by several IS from *wbkC* (BMEI1418), was identified before as a LPS gene in *B. abortus* [19] and encodes a 335 amino acid protein with homology to polyisoprenyl-phosphate N-acetylhexosamine-1-phosphate transferases (PNPT). Known PNPTs include *Pseudomonas aeruginosa* PAO1 WbpL and *Escherichia coli* WecA, both involved in bactoprenol priming but belonging to two subfamilies that differ in aminosugar specificity [20]. A search for WbpL (339 amino acids) homologues in *B. melitensis* identified BMEI1426 as the closest one (BLAST E-value = 4e^−22^). For WecA (367 amino acids), not only BMEI1426 (1e10^−8^) but also BMEII0839 (1e10^−42^) were identified. However, the only WecA match in *B. abortus* genomes was the BMEI1426 ortholog (BruAb1_0533 and BAB1_05359-49 in *B. abortus* 9-49 and 2308, respectively). This result, which is due to a large deletion in *B. abortus* chromosome II encompassing the position of the BMEII0839 ortholog [21], supports the role of BMEI1426 as the *Brucella* PNPT gene. The BMEI1426 protein resembled WecA and WbpL in the Asp rich consensus motif of the catalytic site [20] but did not show the motifs thought to relate to substrate specificity in either subfamily. Since it could not be ascribed to any of these subfamilies, we named this gene *wbkF*. Finally, we named the adjacent ORF (BMEI1427) *wbkD*. The protein is annotated as an epimerase/dehydratase and carries a NAD or NADP binding motif, two features of the trifunctional UDP-N- d-acetylglucosamine 4,6- dehydratase/5-epimerase/3-epimerase enzymes that take part in the synthesis of 2-acetamido-2,6-dideoxy-L-hexoses such as N-acetylfucosamine and N-acetylquinovosamine [22]. Quinovosamine (2-amino-2,6-dideoxy-D-glucose) has been reported in the LPS of S *Brucella* spp. [23]–[27] but its location is unclear. Since N-acetylquinovosamine synthesis would require an epimerase/dehydratase using NADP as the coenzyme [22], we investigated the S-LPS hydrolytic polysaccharides of *B. melitensis* and *B. abortus*. In addition to the N-formylperosamine signals, a resonance at 2.06 ppm. indicated the presence of an N-acetyl group in these materials. The integrals of these signals compared to those from the methyl groups of perosamine at ∼1.3 ppm showed that the former derived from ∼1% of the material. Moreover, the ^1^H NMR spectrum at 25°C of the *B. abortus* material (which was highly pure) showed a resonance at 4.56 ppm having JH1,H2 = 7.9 Hz. Further analysis was performed at 70°C (Figure S2) using in particular 2D ^1^H, ^1^H- total correlation spectroscopy (TOCSY) with spin-lock times up to 120 ms by which a complete spin-system corresponding to a quinovosamine residue was traced out. The following chemical shifts were identified: δ 4.54 (H1), 3.79 (H2), 3.66 (H3), 3.35 (H4), 3.50 (H5), and 1.30 (H6). Thus, the analysis revealed the characteristic ^1^H NMR chemical shifts of a β-linked quinovosamine, which should be N-acetylated and is presumably 3-substituted [28], [29]. Indeed, this N-acetylquinovosamine could be the substrate of the putative PNPT WbkF, since WbkF is close to WbpL and the latter transfers either N-acetylfucosamine or N-acetylquinovosamine [20]. Moreover, the adjacent position of *wbkF* and *wbkD* also suggests a functional connection.

Extension of the *wbk* region up to BMEI1393 (*wbkE*) (Figure 2B) suggested that BMEI1394, BMEI1395 and BMEI1396 (putative mannose-6-phosphate isomerase [*manA*], mannose-1-phosphate guanylyltransferase [*manC*] and phosphomannomutase [*manB*]) could be involved in the synthesis of mannose, the precursor of perosamine (Figure 2A). However, when we disrupted BMEI1396 (*manB*), the mutant still expressed O-PS demonstrating that the gene was not essential for mannose synthesis.

#### Mutants affected in core oligosaccharide synthesis

Mutant BMEI1326 had a R2 LPS like that observed before in a *B. abortus* orthologous mutant [11], and encodes a predicted glycosyltransferase of family 25. This is worth mentioning because this family contains LPS glycosyltransferases, including the WaaX protein that takes part in the synthesis of some *E. coli* core chemotypes [30]. BMEI1326 is isolated from other LPS genes and, in the absence of more information, we maintained its provisional name (wa**) [11]. Mutant in BMEI1886 was affected in a putative phosphoglucomutase (Pgm) and had a R2 LPS (Figure 2), in agreement with a previous observation in *B. abortus* [31] and with the presence of glucose in the *Brucella* LPS core [26]. Finally, BMEII0899 disruption generated a R3 LPS (Figure 2) like the one reported for the orthologous *B. abortus* mutants [11], [19]. It is annotated as phosphomannomutase gene and, because of the severe core defect, we proposed before the name of *manB_core_* [11]. However, taking into account the lack of R phenotype in the mutant in the *manB* gene of region *wbk* (see above) and the absence of additional *manB* annotations in *Brucella*, *manB_core_* plus the contiguous *manC* seem the only genes of the pathway providing mannose for both perosamine and core synthesis (Figure 2A).

#### Mutants blocked in O-PS export

**Figure 3:**
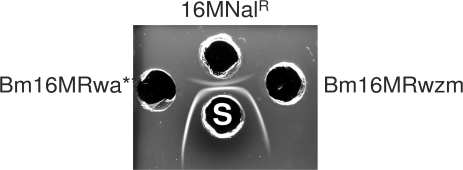
*B. melitensis* mutants in genes wa** and *wzm* synthesize N-formylperosamine polysaccharides. The figure shows a gel immunodiffusion analysis of the polysaccharides obtained from mutants Bm16MR*w*a** and Bm16MR*wzm*. Well 16MNal^R^ contained the LPS of the parental strain and shows both the slow diffusing S-LPS and the fast diffusing native hapten polysaccharide precipitin lines; (S), serum from naturally infected cattle.

To study whether the R mutants could elicit antibodies to the O-PS, we infected mice and tested the sera in an enzyme-linked immunosorbent assay with *Brucella* native hapten, a N-formylperosamine polysaccharide that lacks core sugars [32]. We observed reactivity in the sera of mice infected with 10^8^ or 10^10^ colony forming units (CFU) of the *wzm* (BMEI1415) mutant or with 10^10^ CFU of the wa** (BMEI1326) mutant (0,810 and 0,714 optical density readings, respectively; 1∶25 dilution). However, the antibody levels were lower than those induced by 10^6^ CFU of the 16M Nal^R^ parental strain (1,480 readings for the same dilution). When we tested extracts of these mutants by gel immunodiffusion with sera from *Brucella* infected cattle, we observed a component giving a reaction of identity with the native hapten polysaccharide (Figure 3). Moreover, ^1^H-NMR analysis of the extracts showed the signals of α1,2- α1,3-linked N-formylperosamine polysaccharides [17] plus a small signal at 2.06 ppm corresponding to the *N*-acetyl group of an unidentified N-acetylated aminosugar. Upon cell fractionation, the polysaccharides were detected in the envelope and cytosol of the *wzm* and wa** mutants, respectively.

#### Mutant designation

Based on the above analyses, we designated the mutants according to the original strain (Bm16M or BmH38), the phenotype (R) and the LPS gene disrupted (Table 1 and Figure 2).

### The LPS defects alter key topological, physicochemical and biological surface properties of *Brucella*

**Figure 4:**
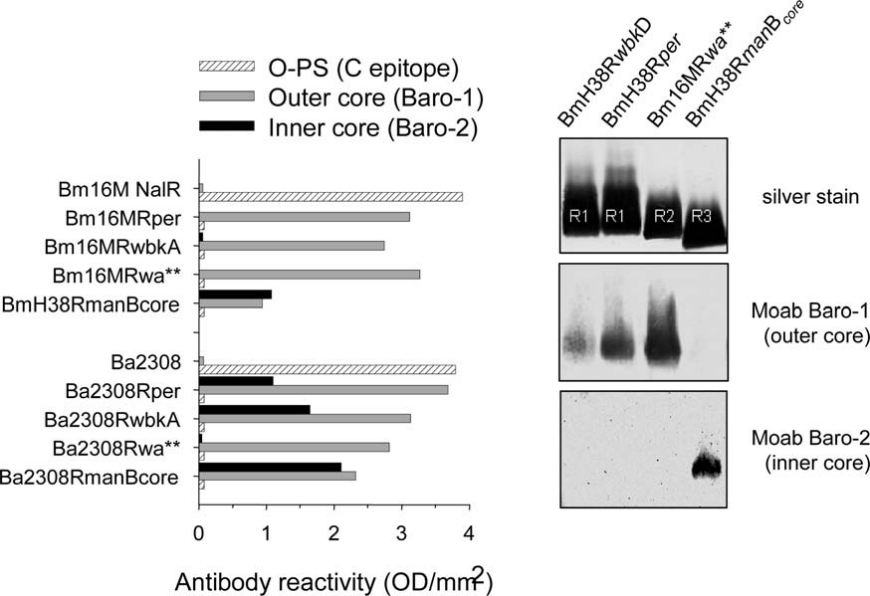
LPS epitopes in smooth *B. abortus* and *B. melitensis* strains and their cognate R mutants. Whole bacteria (left panel) or *B. melitensis* R-LPS representative of the R1, R2 and R3 types (right panel) were probed with monoclonal antibodies of the indicated specificity (Ba2308, *B*. *abortus* 2308; Bm16MNal^R^, *B. melitensis* 16M Nal^R^; codes for the R mutants are those used in the text).

It is known that outer membrane proteins (Omp) are more exposed on R than on S brucellae [11], [33], and we confirmed this for R1, R2, and R3 mutants using monoclonal antibodies (Moabs) to Omp1, Omp2b, Omp31, Omp25, Omp19, Omp16 and Omp10 (not shown). Less is know about the topology of R-LPS epitopes and, therefore, we tested the mutants with the appropriate Moabs. In all cases, absence of O-PS correlated with exposure of the outer core (Figure 4, left panel) and the lipid A disaccharide (Moab Bala-1; not shown). Surprisingly, the R1 and R2 but not the R3 mutants failed to react with the inner core Moab Baro-2 (Figure 4, left panel). Since Baro-2 was produced by immunization with *B. abortus* [34], we confirmed its reactivity using *B. abortus* 2308 mutants in *per* (Ba2308R*per*), *wbkA* (Ba2308R*wbkA*), wa** (Ba2308Rwa**) and *manB_core_* (Ba2308R *manB_core_*) (Figure 4 left panel). As expected, all these R mutants but wa** [11] reacted with Baro-2. Then, we tested representative *B. melitensis* R-LPS by Western blot. The LPS of BmH38R*wbkD* (R1), Bm16MR*per* (R1) and Bm16MRwa** (R2) but not that of BmH38R*manB*_core_ (R3) reacted with Baro-1 (Figure 4, right panel). Conversely, Baro-2 failed to detect an inner core epitope in the R1 and R2 but not in the R3 LPS (Figure 4, right panel). These results show hitherto undescribed LPS core differences between *B. abortus* and *B. melitensis*.

**Figure 5:**
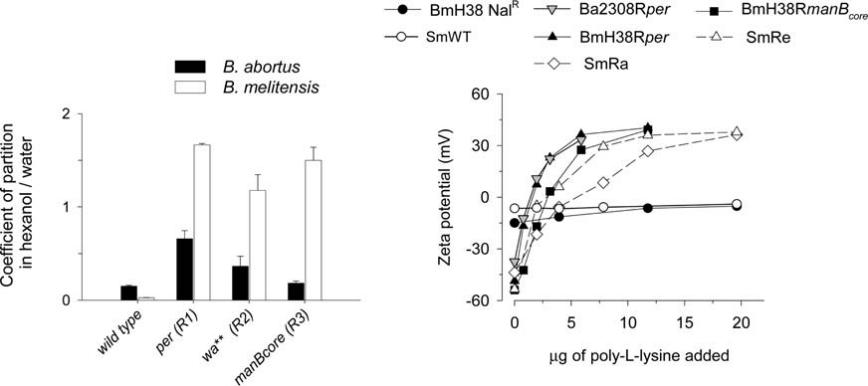
Surface hydrophobicity (partition in hexanol/water) and charge (zeta potential in dependence of poly-L-lysine) of smooth *B. abortus* and *B. melitensis* parental strains and their cognate R mutants. For *Brucella*, the genes (left panel) or the strain codes (right panel) indicated are those used in the text. *S. minnesota* controls were: wild type, SmWT; Ra LPS mutant, SmRa; Re LPS mutant, SmRe.

All R mutants displayed an increased surface hydrophobicity, more markedly in *B. melitensis* than in *B. abortus* (Figure 5, left panel), and also in sensitivity to the polycationic lipopeptide polymyxin B (not shown). The latter effect was explained by the increase in negative Zeta potential (surface charge) of the R mutants (Figure 5 right panel). However, we did not observe a clear correlation between Zeta potential and LPS defects (−58 to −64 mV for R1, −55 to −58 mV for R2, and −66 to −68 mV for R3), like that obtained for the *S. minnesota* R chemotypes (Ra, −39 mV; Rc, −44 mV, and Re, −53 mV). Since the LPS core is complete in Ra, lacks the distal N-acetyl-glucosamine in Rc, and is reduced to two Kdo residues in Re, the comparison shows that the outer core does not balance the negative charge of the Kdo-lipid A section in *Brucella* LPS. In support of this interpretation, all *Brucella* R mutants behaved like the *S. minnesota* Re mutant when the Zeta potential was measured in the presence of the polycation poly-L-lysine (Figure 5, right panel). The Zeta potential value and the polycation effects were clearly reduced in the S parental strains *S. minnesota* HL63 and *B. melitensis* H38 Nal^R^ (Figure 5, right panel) or *B. melitensis* 16M Nal^R^ (not shown).

**Figure 6:**
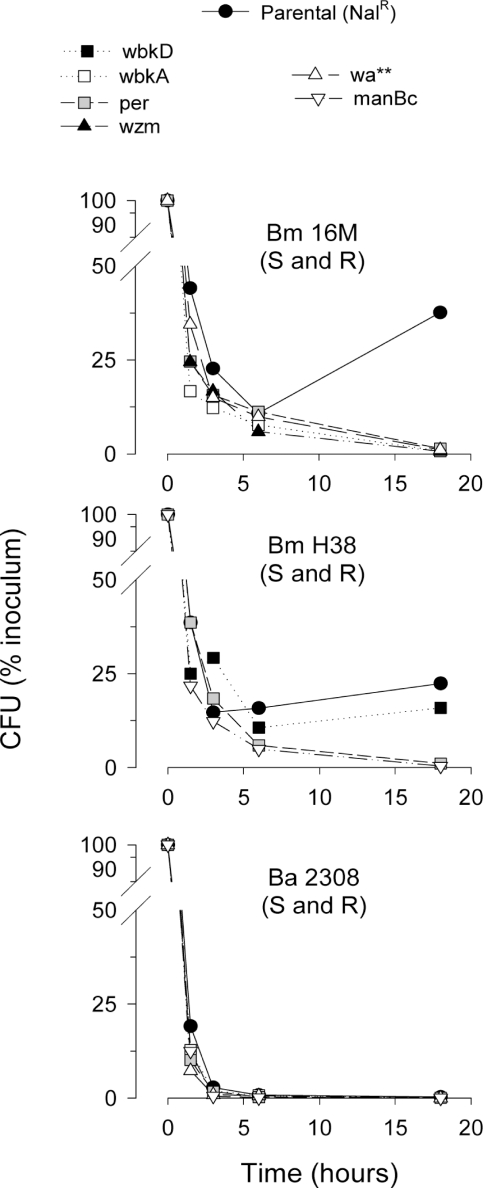
Survival of smooth *B. melitensis* and *B. abortus* parental strains and their cognate R mutants in normal ovine serum.

Resistance to complement-mediated killing in normal serum is a biologically significant property of S brucellae [35]. We found that, whereas the *B. melitensis* S strains were only partially affected by normal sheep (Figure 6) or cattle serum (not shown), all *B. melitensis* R mutants but BmH38R*wbkD* were killed within 20 hours. Interestingly, neither the *B. abortus* 2308 Nal^R^ S strain nor the cognate R mutants survived under the same conditions (Figure 6).

### R mutants show different patterns of attenuation

**Figure 7:**
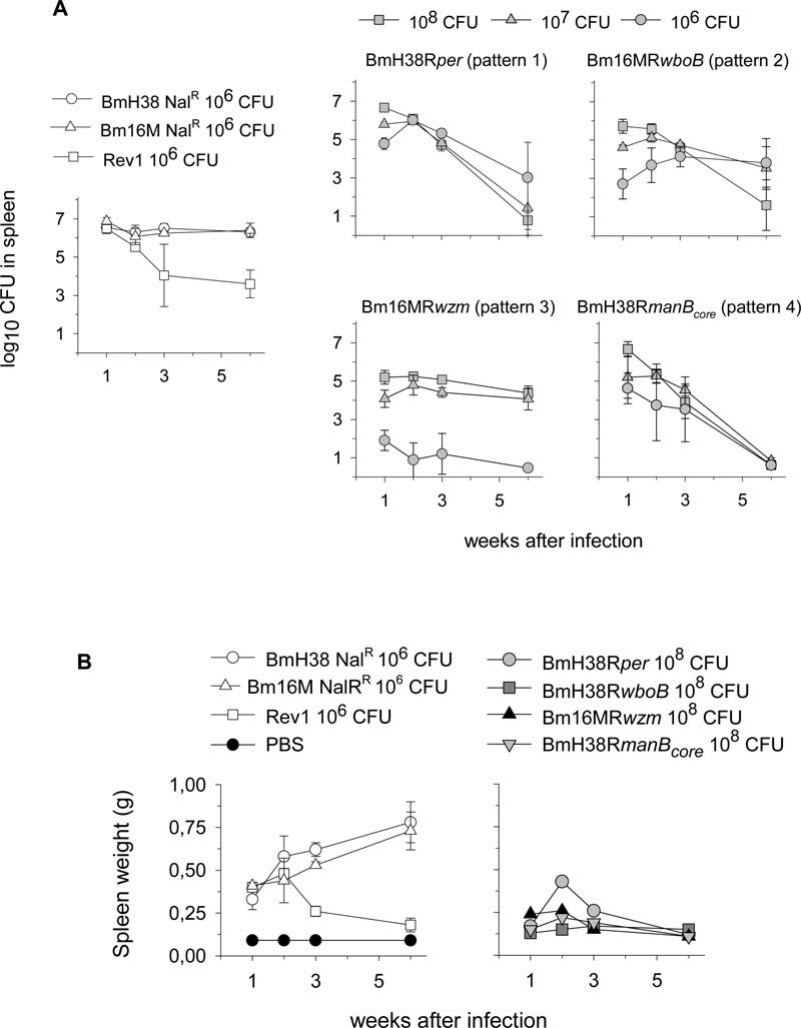
Attenuation patterns of *B. melitensis* R mutants and vaccine Rev 1 in BALB/c mice in comparison with smooth *B. melitensis* parental strains . Panel A, evolution of CFU/spleen; panel B, spleen weights.

To assess attenuation, we inoculated BALB/c mice with three doses of each R mutant or with 10^6^ CFU of the parental strains and vaccine Rev 1, and determined the CFU numbers in the spleens. All R mutants were less persistent than the parental strains, with differences that allowed classifying them into four broad attenuation patterns (Figure 7A). Pattern 1 comprised BmH38R*per*, BmH38R*wbkD* and BmH38R*wbkF*, all of the R1 type. They multiplied at 10^6^ CFU/mouse and, no matter the dose, reached spleen counts at week 2 similar to those of the parental strain. Then, the CFU decreased markedly. Pattern 2 included BmH38R*wboA*, Bm16MR*wboB*, Bm16MR*wbkE*, BmH38R*wbkE*, Bm16MR*wbkA*, Bm16MR*gmd*, Bm16MR*per* (all R1) and Bm16MR*pgm* (R2). Although able to replicate, they never reached the parental strain level and were cleared at rates directly related to the inoculum size. Pattern 3 included Bm16MR*wzm* and Bm16MRwa**, two mutants with internal N-formylperosamine polysaccharides and reduced *in vitro* growth rates. Although not multiplying when inoculated at 10^6^ CFU, they persisted at relatively high numbers at the end of the experiment (week 6) for the 10^7^ or 10^8^ CFU doses. To further examine their persistence, we inoculated mice with 10^8^ CFU/mouse and examined them 6, 9 and 12 weeks after infection. The CFU/spleen at week 6 were similar to those of the first experiment, declined more than 2 logs at week 9 and the mutants were cleared by week 12. Remarkably, the results of pattern 3 mutants (10^7^ or 10^8^ CFU) and Rev 1 almost overlapped at weeks 2, 3, 6 (Figure 7), 9 and 12 (not shown). Finally, we separated BmH38R*manB_core_* (R3 and accelerated growth *in vitro*) into pattern 4 because its clearance started as early as week 2 and was complete between weeks 3 and 6.

As reported before [36], splenomegaly caused by virulent *Brucella* increased throughout the experiment (Figure 7B). In contrast, the weights of the spleens of Rev 1 inoculated mice peaked at week 2 and declined afterwards. Only pattern 1 R mutants inoculated with 10^8^ CFU induced transient splenomegaly similar to that of Rev 1 (Figure 7B).

### Some R mutants reach the *Brucella* replicative niche in macrophages

**Figure 8:**
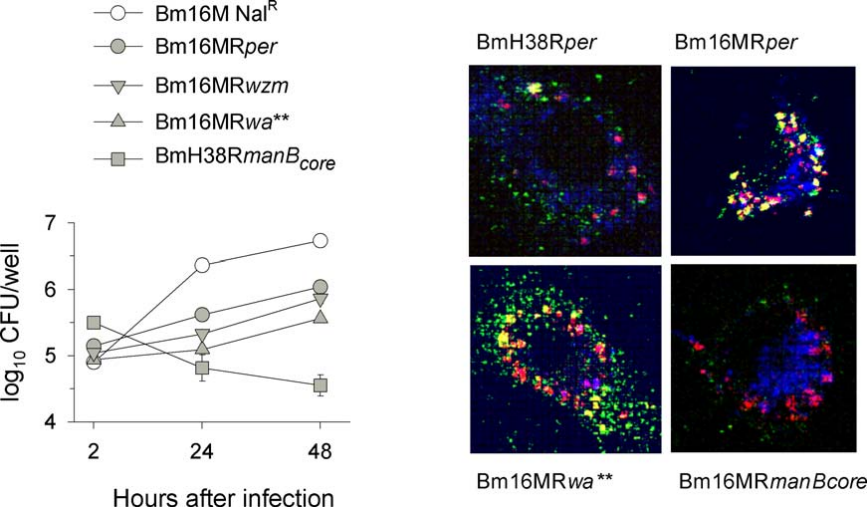
Multiplication and intracellular localization in BMDM of representative *B. melitensis* R mutants. Left panel, evolution of intracellular bacteria CFU numbers. Right panel, colocalization of selected R mutants (immunostained in red) with LAMP1 (immunostained in blue) or calreticulin (immunostained in green) in BMDM.

Since a brucellosis vaccine must persist long enough to trigger protective immunity, it has to be able to multiply intracellulary. Thus, we studied the multiplication of BmH38R*per*, Bm16MR*per* and (patterns 1 and 2, both R1), Bm16MR*wzm* (pattern 3, R1 and internal N-formylperosamine polysaccharides), Bm16MRwa** (pattern 3, R2 and internal N-formylperosamine polysaccharides) and BmH38R*manB_core_* (pattern 4, R3) in bone-marrow derived macrophages (BMDM). We found that only BmH38R*manB_core_* was unable to grow in BMDM, and that no mutant was as efficient as the virulent strain (Figure 8, left panel). We also examined the intracellular fate of representative mutants. After 24 hours, some bacteria of patterns 1, 2 and 3 were in calreticulin (endoplasmic reticulum marker)-positive but also in LAMP1-positive compartments. At this time, pattern 3 bacteria were in comparatively high amounts in the calreticulin-positive compartments of some BMDM and BmH38R*manB_core_* was completely degraded in LAMP1-positive compartments (Figure 8). To extend these observations, we determined the location of mutants BmH38R*wbkF*, BmH38R*wbkD* (both of pattern 1), Bm16MRwa** (pattern 3), and BmH38R*manB_core_* (pattern 4) 0.5, 2, 4, 8, 12, and 24 hours after infection. For the latter, colocalization with LAMP1 was observed 0.5 hours after infection, and full destruction by the 4th hour. BmH38R*wbkD* and BmH38R*wbkF* were quickly in LAMP1-positive compartments but intact bacteria persisted up to 8 to12 hours and some reached calreticulin-positive compartments. Bm16MR1.17wa** also transited through LAMP1-positive compartments and then co-localized efficiently with calreticulin, persisting intact at least 24 hours.

### R mutants can be ranked by their ability to immunize against virulent bacteria

Using BmH38R*wbkD* (pattern 1), Bm16MRwa** (pattern 3), and BmH38R*manB_core_* (pattern 4), we determined the conditions necessary to rank R mutants by their ability to induce protection by the most favorable (i.e. intraperitoneal) route [8]. We studied the vaccine dose (from 10^6^ to 10^8^ CFU), two strains of mice (BALB/c and CD-1), the vaccination-challenge interval (4 and 8 weeks), and the challenge dose (10^4^ and 10^5^ CFU) and strain (16M and H38). A vaccine dose below 10^8^ CFU or a 10^5^ CFU challenge resulted in poor or no protection, and a vaccination-challenge interval of 8 weeks did not improve the evaluation. Similarly, there was no influence of the challenge strain regardless of the 16M or H38 background of the mutants. Finally, the *Brucella*-susceptible BALB/c mice yielded a larger span between control groups (PBS [150 mM NaCl, 7 mM KH_2_PO_4_, 10 mM K_2_HPO_4_; pH 6.85]- and Rev 1-inoculated mice) and more homogenous results than CD-1 mice, thereby allowing better statistical evaluations (see Figure S3).

Then, we performed a first experiment (Table 2) which ranked the 14 R mutants into three sets. The first one (Bm16MRwa**, Bm16MR*wzm*, BmH38R*wbkF*, BmH38R*wbkD*, and BmH38R*per*) generated protection not significantly different from that provided by Rev 1 (at the dose and by the route used as reference in mice). A second set (BmH38R*wboA*, Bm16MR*pgm*, and Bm16MR*gmd*) generated protection but less than Rev 1, and the third set (6 mutants) failed to protect. For a better discrimination, we tested the first set of mutants (plus two mutants of the third set as references) using 10 mice per group (Table 2). The results confirmed Bm16MRwa**, Bm16MR*wzm* and BmH38R*wbkF* as the best R vaccines. However, protection by BmH38R*per* and BmH38R*wbk*D was less than that by Rev 1.

**Table 2:** Protective efficacy against *B. melitensis* of *B. melitensis* R mutants (10^8^ CFU) administered intraperitoneally.

			Experiment 1 (5 mice per group)		Experiment 2 (10 mice per group)	
Vaccine	Type of LPS	Attenuation pattern	Log_10_ CFU in spleen (X±SD)	Units of protection a	Log_10_ CFU in spleen (X±SD)	Units of protection a
Bm16MRwa**	R2	3	2.07±1.33 b, c	3.90	1.83±1.23 b, c	4.37
Bm16MR*wzm*	R1	3	2.65±1.88 b, c	3.32	2.48±1.73 b, c	3.72
BmH38R*wbkF*	R1	1	2.91±0.87 b, c	3.06	3.32±1.50 b, c	2.88
BmH38R*wbk*D	R1	1	3.40±1.51 b, c	2.57	3.76±1.41 b, d	2.44
BmH38R*per*	R1	1	3.70±1.72 b, c	2.27	4.10±0.82 b, d	2.10
BmH38R*wbo*A	R1	2	4.31±0.60 b, d	1.66		
Bm16MR*pgm*	R2	2	4.62±0.56 b, d	1.35		
Bm16MR*gmd*	R1	2	4.72±0.50 b, d	1.25		
BmH38R*wbk*E	R1	2	4.99±0.73 d	0.98		
Bm16MR*wbk*A	R1	2	5.05±0.69 d	0.92		
Bm16MR*wbk*E	R1	2	5.06±0.43 d	0.91		
Bm16MR*wbo*B	R1	2	5.38±0.36 d	0.59		
Bm16MR*per*	R1	2	5.38±0.70 d	0.59	5.44±0.51 d	0.76
BmH38R*manB_core_*	R3	4	4.88±0.57 d	1.09	5.49±0.52 d	0.71
Rev 1 e	S		2.76±0.44 b	3.21	2.63±0.90 b	3.57
PBS			5.97±0.19		6.20±0.08	

a Average of log_10_ CFU in the spleens of saline inoculated mice minus average of log_10_ CFU in the spleens of vaccinated mice.

b P<0.005 versus PBS.

c P>0.05 versus Rev 1 vaccinated.

d P<0.005 versus Rev 1 vaccinated.

e 10^5^ CFU subcutaneously.

### Only attenuation pattern 3 R mutants approach Rev 1 as *B. melitensis* vaccines

Intraperitoneal inoculation is unpractical in the natural host. Thus, we tested the efficacy of the five best R mutants when administered subcutaneously. As shown in Table 3, only pattern 3 mutants compared to Rev 1 under these conditions. However, the dose used was one thousand fold higher than that of Rev 1 (10^8^ and 10^5^ CFU, respectively). Higher doses of these R mutants generated abscesses and other untoward effects.

**Table 3:** Protective efficacy against *B. melitensis* of selected *B. melitensis* R mutants administered subcutaneously.

Vaccine (dose)	Log_10_ CFU in spleen (X±SD)	Units of protection a
Bm16MRwa** (10^8^ CFU)	3.19±0.63 b, c	3.10
Bm16MR*wzm* (10^8^ CFU)	2.22±1.48 b, c	4.07
BmH38R*wbkF* (10^8^ CFU)	4.92±0.51 b, d	1.37
BmH38R*wbk*D (10^8^ CFU)	5.08±0.54 b, d	1.21
BmH38R*per* (10^8^ CFU)	4.96±0.67 b, d	1.33
Rev 1 control (10^5^ CFU)	3.02±1.01 b	3.27
PBS	6.29±0.15	-

a Average of log_10_ CFU in the spleens of saline inoculated mice minus average of log_10_ CFU in the spleens of vaccinated mice.

b P<0.005 versus PBS.

c P>0.05 versus Rev 1 vaccinated.

d P<0.005 versus Rev 1 vaccinated.

## Discussion

We present here a comprehensive study aimed to clarify the value of brucellosis R vaccines that is based on an understanding of the genetics of *Brucella* LPS synthesis, rather than on empirical approaches. LPS synthesis is known to occur through three main pathways that lead to the synthesis of the O-PS, core oligosaccharide and lipid A [37]. Some steps of the last two pathways overlap to generate the (Kdo)_2_- lipid IVa precursor on which sugars other than Kdo are then assembled to generate a complete R-LPS (R1 in the classification used in the present work). Accordingly, deficiencies in these processes generate deeper R (R2 and R3) phenotypes than those affecting O-PS synthesis. Polymerization of the latter takes place on the bactoprenol carrier which subsequently shuttles the polymer to the periplasm, the place where it is linked to R1-type R-LPS. Therefore, mutants in O-PS synthesis generate R1 phenotypes which may be accompanied or not by accumulation of O-PS precursors and may have side effects on other envelope located functions. In addition to the above-summarized pathways, there are ancillary routes providing the core and O-PS building units. Genomic surveys show that the three main pathways are present in *Brucella* [38], and the mutations studied here affect key steps of the O-PS and core pathways as well as the synthesis of precursors.

Although we screened about 16,500 transposon mutants from *B. melitensis* H38 Nal^R^ and 16M Nal^R^, and expanded the investigation to those ORFs with suggestive annotations that flanked some eventually spurious R mutants, we only found one gene (*wbkD*) that, to the best of our knowledge, had not been identified as a LPS gene or as a virulence-related gene [11], [16], [19], [24], [39]–[41]. On this basis, it seems that only the *wbk* (Figure 2B) and *wbo* regions encode proteins dedicated to *Brucella* O-PS synthesis. Intriguingly, *wbk* contains several genes that, upon disruption, do not generate R phenotypes. They include *wbkB* [16] and BMEI1396, annotated as *manB*. We proposed before that the putative *man* genes in *wbk* could be involved in O-PS synthesis [11] but the evidence that disruption of BMEI1396 fails to generate a R phenotype is against this hypothesis. It is still possible that *manB*_core_ (BMEII0899) could internally complement the defect in the *wbk manB* mutant. However, since *manB*_core_ mutants show a deep R phenotype, it would remain to be explained why the converse internal complementation is not effective. Studies on the expression and activity of the proteins encoded by the putative *man* genes in *wbk* are necessary for a definite conclusion. The *wbk* region also contains the ABC transporter genes (*wzm* and *wzt*) typical of homopolymeric O-PS, and the implication of *wzm* and *wzt* in O-PS export was established in a previous work [16]. We extended this demonstration by purifying the N-formylperosamine polysaccharide built up by Bm16MR*wzm* and showing its location in the cell envelope, in all likelihood bound to bactoprenol, a linkage that could overcome its haptenic nature and account for its immunogenicity. In contrast, the polysaccharide built up by mutant BmR16MRwa** was found mostly in the soluble fraction. This suggests a transient bactoprenol linked state and, consistent with the R2 LPS pattern, it may be that Wa** transfers a sugar necessary for the linkage of the O-PS to the core. This step occurs in the periplasm [37], but our results are not in disagreement with this because the physical fractionation methods used do not allow to distinguish periplasmic and cytosoluble fractions in *Brucella*. Interestingly, both the wa** and the *wzm* mutants showed delayed generation times consistent with a linkage of the O-PS precursor to bactoprenol and a reduced availability of the latter for other envelope biogenesis processes. The retarded growth of mutant *pgm*, however, can be explained by the role of Pgm in the metabolic steps that use glucose-nucleotides. Obviously, it can be reasoned that the *manB*_core_ mutant owes its accelerated growth to absence of O-PS synthesis. These differences in growth rates are relevant traits in a live vaccine candidate.

The attenuation of R *Brucella* mutants has been known for decades [3] and it evidently relates to changes in the bacterial surface that affect the interaction of bacteria with cells and soluble effectors of the immune system. Concerning the host cells, it has been observed that R mutants penetrate more actively and display higher adherence to nonphagocytic and phagocytic cells than S *Brucella* [42]–[48]. In addition, Porte et al. [39] found that, whereas virulent *B. suis* cells select lipid rafts to enter into murine macrophages, R *B. suis manB_core_* cells do not, hence showing the critical role of the O-PS at the port of entry. This role could be that of a negative modulator of non specific adherence which would allow receptors to act, that of the ligand of an unknown cell receptor, or both [39], [41]. Indeed, a definition of the nature of the surface changes should contribute to better understand this key aspect of *Brucella* virulence. Despite this, only one study conducted with the spontaneous R mutant *B. abortus* 45/20 has partially addressed the overall effects of the O-PS deficiency [49]. Here, we demonstrate that the surface of the *Brucella* outer membrane, once devoid of the O-PS, becomes simultaneously highly hydrophobic and negatively charged, and that the negative charge relates exclusively to inner core-lipid A groups. That this negative charge was similarly abrogated by the O-PS in *S. minnesota* HL63 and S *Brucella* leads to the conclusion that we are dealing with general physicochemical properties not linked to the particular sugar (N-formylperosamine) in S *Brucella* O-PS. Accordingly, the overall physicochemical picture for the R mutant surface is that of a mosaic of negative charges scattered among hydrophobic spots. Such a surface should both allow and bring about multiple non specific interactions with eukaryotic membranes that should override any specific binding. This picture is in keeping with the observations on the invasiveness and attachment of R *Brucella* mutants and supports the hypothesis that the O-PS acts at least in part as a negative modulator. Clearly, the loss of the ability to select the port of entry and the possible unspecific interactions with the membranes of the internal compartments where *Brucella* multiplies are factors that can contribute to attenuation.

Concerning the surface topological changes, we confirmed the differences in Omp and lipid A epitope exposure existing between R and S bacteria [11], [33]. However, the differences in LPS core epitopes observed between *B. abortus* and *B. melitensis* were unexpected. This divergence adds to the absence of Omp31 and possible surface elements related to the 25 Kb deletion of *B. abortus* chromosome II [21], and both have to be considered in the light of other cell surface properties. Over 40 years ago, it was observed that normal human serum has a more powerful killing activity on *B. abortus* than on *B. melitensis* or *B. suis* and that R *Brucella* mutants are cytophatic for monocytes, a phenomenon that would leave the invaders exposed to the bactericidal action of serum [3], [50]–[52]. These findings and hypothesis have been updated in recent works. Fernandez-Prada et al. [53] noted that *B. abortus* 2308 *wboA* mutants (R1 according to our results) are more sensitive to normal human serum than homologous *B. melitensis* 16M mutants. This species difference is supported and extended by our experiments with the R1, R2, and R3 mutants from *B. abortus* 2308 Nal^R^, *B. melitensis* H38 Nal^R^ and 16M Nal^R^ and sheep and cattle sera. Moreover, since the difference persisted in the *B. abortus* and *B. melitensis manB_core_* mutants (which display similar core stubs), it is unlikely that core variations in *B. abortus* and *B. melitensis* could account for it. Therefore, it seems that the absences of Omp31 and possible surface elements related to the 25Kb deletion in *B. abortus* [21] account for the greater sensitivity to normal serum, as proposed by Fernandez-Prada et al. [53]. These same authors also found that *B. melitensis* 16M and its *wboA* R mutant are resistant to normal human serum. In our conditions, however, only one of the seven R mutants (BmH38R*wbkD*) survived, indicating that complement sensitivity is a usual characteristic of *B. melitensis* R mutants whose contribution to attenuation cannot be dismissed.

Interestingly, not all R mutants were equivalent in surface properties, suggesting that they could display different degrees of cell interaction and virulence which would allow the selection of optimal vaccines. Assessment of this point required appropriate laboratory models and we used both animals and cultured cells. The animal used in the vast majority of works evaluating *Brucella* virulence is the mouse, and spleen CFU is the main criterion. However, literature perusal shows that mouse studies with R mutants vary in infectious dose from 10^4^ to 10^8^ CFU [8]. Preliminary observations and some apparently conflicting results reported in the literature led us to test the possibility that a combination of doses could yield better analyses. This was proved correct and we classified the mutants into four broad patterns. Pattern 1 mutants inoculated at the lower dose multiplied transitorily reaching spleen counts similar to those of Rev 1 and the S virulent strains. Consistent with this, they reached compartments bearing the endoplasmic reticulum marker calreticulin and multiplied there. These R mutants were the only ones inducing a splenomegaly similar to that of Rev 1. Since splenomegaly correlates with IFN-γ and IL-12 levels in mouse brucellosis and both cytokines are decisive for mounting effective immunoresponses to *Brucella* [54], it seemed that pattern 1 mutants stimulated immunity better than other R mutants. This interpretation was born out by the ranking of the mutants by their immunizing ability. Despite this, pattern 1 mutants were not equivalent to Rev 1 because the latter strain persisted longer in spleens. Concerning pattern 2, a remarkable feature was a conversely related dose-persistence relationship, a trend that, although less clearly, was also perceived in pattern 1. It has been shown that *Brucella* behaves as a stealthy parasite that avoids detection by innate immunity at the onset of infection, thus retarding an adaptive cellular response and making possible for the parasite to reach sheltered intracellular niches [35]. This ability is not related to the induction of regulatory cytokines such as IL-10 but rather to a reduction in the pathogen-associated molecular pattern of those envelope molecules (most notably LPS) normally recognized by TLR bearing cells [35]. According to this, it can be predicted that mutants in LPS, a molecule critical in avoiding innate immunity by *Brucella*, could elicit innate immunity (and hence cellular immunity) in a dose-dependent fashion and be thus eliminated faster at higher than at lower doses. Indeed, our results are consistent with this prediction. Concerning pattern 3 mutants, it can be proposed that their longer generation times account for the lower CFU in spleen and, in keeping with the *Brucella* stealthy strategy, for a lower stimulation of innate immunity that favors persistence. These mutants did not multiply at the level of the parental strains in BMDM but they were surprisingly efficient in reaching endoplasmic reticulum-derived compartments. Although the first characteristic could relate to their long generation time, we have no clear hypothesis to propose for the second observation. We can speculate that it is somehow connected to the internal O-PS precursor they carry, since these mutants were the only ones that had detectable amounts of it. It has to be considered that a fraction of the pattern 3 bacteria was destroyed by BMDM, and that this should release the O-PS precursor within the cell. The effect of free N-formylperosamine polysaccharides within the *Brucella* containing vacuoles has not been studied and, in the light of our results and of the presence of these free polysaccharides in S brucellae [32], this is an aspect that deserves attention. Finally, the marked attenuation of the pattern 4 mutant was evident in all experiments and in keeping with the notion [11] that a severe core LPS damage abrogates useful immunogenicity.

It is interesting that all pattern 1 mutants derived from *B. melitensis* H38 and that some homologous ones in *B. melitensis* 16M (like Bm16MR*per*) belonged to pattern 2 and were poor vaccines. These mutants behaved similarly in BMDM, suggesting that early events in host cells are not the reason for the differences in mice. It seems, therefore, that the overall genetic background of the parental strains is relevant and that selection of *Brucella* vaccines cannot be based only on the identification of a target gene whose dysfunction generates attenuation. In this regard, it is worth commenting that the literature reveals that strain16M stocks sometimes yield CFU/spleen lower that those obtained in our experiments with either H38 or our 16M. We have tested the virulence of 16M obtained from several laboratories and found that, in fact, some were less virulent than others, suggesting that the stability of this strain cannot be taken for granted (M.J. Grilló, and J.M. Blasco, unpublished results).

Like virulence studies, the laboratory assessment of brucellosis vaccines has been almost always based on experiments in mice, and there is a useful mouse model for the evaluation of S19 and Rev 1 vaccine stocks. This model uses a tight set of conditions chosen to reflect in mice field observations on residual virulence and immunogenicity [55]. However, there are no studies in the natural hosts with different *Brucella* R vaccines. Therefore, one aim of our work was to set up a model to rank R mutants for their ability to induce protection, and we defined some experimental conditions for this. Using these conditions, we found that the top candidates were Bm16MRwa**, Bm16MR*wzm,* BmH38R*wbkF*, BmH38R*wbk*D, and BmH38R*per*. These mutants belonged to patterns 1 and 3, were able to multiply in BMDM and to reach the niche where virulent brucellae replicate, and all but Bm16MRwa** were of the R1 phenotype. This result with Bm16MRwa** is not in contradiction with our previous observation that a complete core was required for maximal efficiency of R *B. abortus* vaccines in mice [11] because Bm16MRwa** (and Bm16MR*wzm*) elicited anti-O-PS antibodies, and these are known to be protective in mouse brucellosis [54]. Therefore, we cannot rule out that priming by the O-PS precursors could contribute to an antibody response to the challenge strain accounting in part for protection. In fact, these two mutants are reminiscent of a *wboA* complemented RB51 construct that produces internal O-PS and that has been shown to generate better protection in mice than RB51 [18]. Also in keeping with the idea that the internal O-PS set the difference with other R mutants, they were the only ones that matched Rev 1 when we assessed subcutaneously, even though at doses one thousand fold higher. Therefore, our results lead to the conclusion that no R mutant completely devoid of O-PS is equal to Rev 1 in the mouse model. On these grounds, and taking into account the range of genetic defects and properties of the R mutants tested, it seems unlikely that any R vaccine lacking the ability to induce anti S-LPS antibodies could match classical brucellosis vaccines in the natural host. Nevertheless, a definite conclusion requires studies with a definite set of R vaccine candidates in sheep and goats.

## Materials and Methods

### Bacterial strains and culture conditions

*B. melitensis* 16M (S, virulent) is the reference strain of biovar 1 [56] and *B. melitensis* H38 (S, virulent) is a virulent biovar 1 strain [15]. The corresponding Nal^R^ strains were selected on tryptic soy agar containing 25 µg/ml of the antibiotic. *B. abortus* 2308 Nal^R^ (S, virulent) and its R mutants (Ba2308R*per* [formerly Ba 9.49], Ba2308R*wbkA* [Ba 2.17], Ba2308Rwa** [Ba 80.16] and Ba2308R*manB_core_* [Ba55.30]) have been described previously [11]. *B. abortus bvrS*::Tn5 2.13 is an avirulent S strain that carries a normal O-PS but is defective in several outer membrane proteins tightly linked to the LPS [57]. *S. minnesota* HL63 is a S strain and its HL100 Ra, HL105R5 Rc, and HL111R595 Re mutants are bacteria bearing a R-type LPS with intact (Ra), truncated outer core (Rc) or highly defective (deep R) core [58]. All these bacteria were kept in skim milk at −80°C, and aliquots grown on tryptic soy agar or broth when needed (repeated *in vitro* passage was systematically avoided). The reference *B. melitensis* Rev 1 vaccine was originally obtained from the INRA station at Nouzilly (France) and conserved freeze-dried. For routine use, the stocks were rehydrated and cultured only once before preparing the appropriate bacterial suspensions (see below) [56].

### Mutagenesis, ORF identification and complementation analysis

Mini-Tn5 mutagenesis was performed by mating *B. melitensis* 16M Nal^R^ or H38 Nal^R^ with *Escherichia coli* SM10 (λpir) carrying the suicidal plasmid pUT/Km [59], and mutants selected on tryptic soy agar with nalidixic acid (25 µg/ml) and kanamycin (50 µg/ml). R mutants were identified by the crystal violet exclusion test and the lack of reactivity with anti-S-LPS IgG [11]. To test the stability of the mutants, 3×10^3^ CFU of each mutant were first seeded on tryptic soy agar, either plain or supplemented with nalidixic acid (25 µg/ml) or kanamycin (50 µg/ml). Mutants showing no variation in CFU numbers (one-way ANOVA) on these media and a stable R phenotype were inoculated intraperitoneally into 8–10 weeks old BALB/c mice (10^6^ CFU/mouse). Two weeks later, the spleens were seeded on tryptic soy agar and colonies retested for roughness and for the presence of Tn5 by Southern blot. The DNA flanking the mini-Tn5 insertion in the selected mutants was cloned and sequenced [45], and ORFs identified using the database and links at http://urbm59.urbm.fundp.ac.be/7Edharbi/aPAGe/. When the regions adjacent to the mini-Tn-5 included ORFs with annotations suggestive of sugar or polysaccharide synthesis, mutants were generated in *B. melitensis* 16M using a disruptive strategy [60]. To this end, an internal fragment of 300 bp. localized in 5′ part of the ORF was amplified by polymerase chain reaction (PCR) and cloned into the *Eco*RV restriction site of plasmid pSKoriTKan. Constructs were transferred into *B. melitensis* 16M Nal^R^ by conjugation with *E. coli* S17 (λpir), and the recombinant clones were selected on tryptic soy agar with nalidixic acid and kanamycin (see above). Chromosome insertion was assessed either by Southern blot with a probe specific for the kanamycin-resistance cassette, or by PCR using two primers annealing at the start of the disrupted ORF and a primer specific for the pSKoriTKan vector. When necessary, confirmation of the disrupted sequence was achieved by PCR. In frame deletion mutants were constructed by overlapping PCR as described previously [61].

For complementation, the PCR amplified gene was first cloned into plasmid pCR®2.1 (Invitrogen S.A., Barcelona, Spain) using T4 ligase and transformed into *E. coli* TOP10 F'. From this, the construct was subcloned into pBBR1MCS-4, transformed first into *E. coli* Xl1Blue KS+ and then into *E*. *coli* SM10 λ pir for conjugation with the corresponding mutant. Alternatively, the *B. melitensis* ORFeome [62] was used. The appropriate clones were extracted and the ORF was subcloned into plasmid pRH001 [63]. The construct was introduced into the R mutant by mating with *E. coli* S17-1 and the conjugants were selected on tryptic soy agar with nalidixic acid and chloramphenicol.

### Bacteriological characterization

Crystal violet exclusion and sensitivity to S (Tb, Wb, Iz) and R (R/C) brucellaphages were studied as described by Alton *et al.* [56]. Growth curves were obtained simultaneously for all bacteria in a BioScreen C (http://www.bioscreen.fi) instrument. For this purpose, multi-well plates containing 250 µl/well of tryptic soy broth were prepared, and 5×10^6^ CFU of each strain inoculated into triplicate wells. To monitor growth, the optical density at 470 nm (OD_470_) was measured at 10 min intervals.

### LPS and polysaccharide extraction

#### Whole cell LPS

Bacteria (0.5 g wet weight) were extracted with 2% SDS, 60 mM Tris-HCl (pH 6.8) (10 ml), digested with DNase (30 µg), RNase (30 µg) and proteinase K (1.5 mg), the LPS precipitated with isopropanol and analyzed directly by SDS-PAGE [64].

#### Extraction of S- and R-LPS with organic solvents

S-LPS was obtained from the phenol phase of a water-phenol extract, and purified by nuclease and proteinase K digestion and by removal of free lipids [26], [32]. LPS from R mutants was extracted with phenol-chloroform-light petroleum (2∶5∶8) (165 mg of freeze-dried bacteria/ml) [65].

#### Extraction and purification of intracellular polysaccharides

BmR16MR*wzm* and BmR16MRwa** cells were resuspended in 0.5 N trichloroacetic acid (30 g wet weight in 150 ml) and stirred at 4°C overnight. After removal of cell debris (15000× *g* for 15 min), the supernatant was neutralized with NaOH, mixed with 3 volumes of ethanol (24 h at −20°C), the precipitate collected (5000× *g* for 15 min), dialyzed and freeze-dried. This crude extract was chromatographed at room temperature on a Bio-Gel P-10 (Bio-Rad Laboratories S.A., Madrid, Spain) column (Vt 150 ml, Vo 48 ml) in 5 mM phosphate buffered saline (pH 7.2), 0.05% NaN_3_. Fractions (2 ml) were examined for cyclic β-glucans by high performance thin layer chromatography and for immunoreactive polysaccharides by immunodiffusion with sera from *Brucella* infected cattle [32], and those free from glucans were pooled, dialyzed and freeze-dried. In addition, cells were disintegrated in the presence of nucleases in a 40K French Pressure Cell Press (SLM Instruments Inc., Urbana, Ill.) operating at 140 Kg/cm^2^, and the soluble and cell envelope fractions separated by ultracentrifugation (60000× *g*, 2 h). The soluble fractions and crude trichloroacetate envelope extracts were tested for immunoreactive polysaccharides without further purification.

#### S-LPS hydrolytic polysaccharide and native hapten polysaccharide

The S-LPS hydrolytic polysaccharides and the native hapten polysaccharide of *B. melitensis* 16M were prepared as described before [32]. The hydrolytic polysaccharide of *B. abortus* 2.13 S-LPS, which is highly pure due to the absence of group 3 Omp tightly bound to LPS in this *bvrS* mutant [57], was obtained by a similar procedure.

#### LPS and polysaccharide characterization

The Kdo assay, and the SDS-PAGE and Western blot procedures have been described previously [11], [32]. Moabs Baro-1 and Baro-2 recognize the outer and inner core epitopes of *Brucella* LPS, respectively, and Bala-1 recognizes the lipid A disaccharide [34]. Moab 31D2 (Ingenasa, S.A., Madrid, Spain) is specific for the C-epitope of the S-LPS. ^1^H-NMR spectra were recorded at 25 and 70°C in D_2_O solution using Varian Inova 600 and 800 spectrometers equipped with 5 mm PFG triple-resonance probes. Chemical shifts are reported in ppm. using external sodium 3-trimethylsilyl-(2,2,3,3-^2^H_4_)-propanoate (TSP, δ_H_ 0.00). Data processing was performed using vendor-supplied software. TOCSY [66] experiments with mixing times of 10, 30, and 120 ms were used for assignment of resonances.

### Surface hydrophobicity and Zeta potential

To assess cell surface hydrophobicity [67], stationary phase bacteria were inactivated in 0.5% NaN_3_ at 37°C overnight, washed twice with 97 mM K_2_HPO_4_, 53 mM KH_2_PO_4_, 0.8 mM MgSO_4_, 21 mM urea, and adjusted to an OD_470_ of 1.0. Equal volumes of this suspension and n-hexadecane were mixed, stirred briefly, incubated at room temperature for 15 min, and the partition coefficient (1- [DO_470_ water phase)/DO_470_ n-hexadecane]) calculated. The surface charge density was measured as the elec­trophoreti­cally effective potential (Zeta potential) [68]. For this, bacteria were inactivated with 0.5% phenol, washed and resuspended in 1 mM CsCl, 10 mM HEPES 10 mM (pH 7.2) at an OD_600_ of 0.2. Measurements were performed at 25°C in a Zetamaster instrument using the PCS 1.27 software (Malvern Instruments Ltd., Malvern, UK) and the settings of aqueous solutions (viscosity = 1,002 cP; dielectric constant = 80,4), either in plain buffer or in buffer supplemented with poly-L-lysine (MW 14600, Sigma-Aldrich Química, S.A., Madrid, Spain).

### Surface epitope mapping

Epitope exposure was tested by dot blot [11] using the above anti-LPS Moabs and the anti-Omp Moabs Omp10 (A68/07G11/C10), Omp16 (A68/08C03/G03), Omp19 (A76/05C10/A08), Omp25 (Omp25) (A70/06B05/A07, A76/02C12/C11, A68/04B10/F05, A68/07D11/B03 and A68/28G06/C07), Omp31 (A59/10F09/G10), Omp2b (A63/03H02/B01), and Omp1 ([Omp89] A53/10B02/A01) [11], [33], [69]. The reaction was measured (OD per mm^2^) using the Imagemaster system (Pharmacia Biotech, Uppsala, Sweden).

### Sensitivity to non immune serum

Exponentially growing bacteria were adjusted to 10^4^ CFU/ml and then mixed with fresh sheep or cattle normal serum (45 µl of cells plus 90 µl of serum per well) in microtiter type plates in duplicate. After incubation for 1, 3, 6 or 18 h at 37°C h with gentle stirring, brain heart infusion broth (200 µl/well) was added, mixed and 100 µl aliquots plated out. The results were expressed as the % survival with respect to the CFU in the inoculum.

### Animal studies

Female BALB/c mice of 7 weeks of age (Charles River Laboratories, Barcelona, Spain) were housed in the animal building of the CITA laboratory (registration number ES 50297012005) with water and food *ad libitum*. Animals were randomly allotted and acclimated for 1–2 weeks before the start of the experiments. Animal handling and experimental procedures were in accordance with European (DOCE 86/609/EEC), National (RD1201/2005), and Regional (Ley 11/2003) directives, and were supervised by the Ethical Committee of the Institution. To prepare the inocula, bacteria were suspended in PBS and adjusted spectrophotometrically to the appropriate CFU/ml (a suspension with an optical density at 600 nm of 0.170 contained ca. 10^9^ CFU/ml) in the same buffer. In all the experiments, the number of CFU administered was determined retrospectively by culturing triplicate aliquots of each inoculum.

#### Virulence

Groups of 20 BALB/c mice were inoculated intraperitoneally with ca. 10^6^, 10^7^ or 10^8^ CFU/mouse in 0.1 ml of PBS. One, 2, 3, and 6 weeks after inoculation, 5 mice of each group were anaesthetized by CO_2_ inhalation, bled intracardiacally and the spleens removed. For the Bm16MR*wzm* and Bm16MRwa** mutants, additional groups of 15 BALB/c mice were inoculated IP at 10^8^ CFU/mouse, and spleen CFU counts performed 6, 9, and 12 weeks later. Controls received 10^6^ CFU/mouse of *B. melitensis* H38, 16M or Rev 1 by the same route. The spleens were processed [70] to calculate the mean and SD (n = 5) of the log_10_ of CFU per spleen, and the results evaluated using the Fisher's Protected Least Significant Differences test.

#### Antibodies to O-PS

Antibodies were measured in each mouse of the above-described groups. In addition, BALB/c mice (n = 5) were inoculated IP with 10^10^ CFU of the appropriate R mutant and bled 6 weeks after this inoculation. Individual blood samples were taken by intracardiac puncture, incubated at room temperature for 4 hours, centrifuged at 1500 rpm for 10 minutes, and sera collected and frozen at −80°C until use. The antibody response was assessed in an indirect ELISA [70] with the N-formylperosamine native hapten polysaccharide adsorbed to the plates [32]. Sera from non-inoculated mice were used as negative controls.

#### Protection

The following conditions were determined to be optimal to rank R mutants by their ability to generate protection against virulent *B. melitensis*. Groups of 5 BALB/c mice were inoculated intraperitoneally with 10^6^, 10^7^ or 10^8^ CFU/mouse of *B. melitensis* R mutant and challenged 4 weeks later with 1×10^4^ CFU of *B. melitensis* H38 injected by the same route. Two weeks later, the spleens processed for CFU counting [70]. To differentiate the challenging strain from the R mutants persisting in the spleens, samples were plated on both BAB and BAB supplemented with 25 µg/ml of kanamycin, and plates with isolated colonies flooded with a crystal violet-oxalate solution [56]. The results, expressed as the mean and SD of the log_10_ of CFU per spleen of the challenge strain, were analyzed using the Fisher's Protected Least Significant Differences (more than 4 groups) or Bonferroni's test (4 or less groups). In a second experiment, the 5 top ranked mutants plus the 2 mutants inducing the lowest protection were reassessed using 10 mice per group. After ranking, the mouse model of protection was used for evaluating the efficacy of the 5 best R mutants (i.e. Bm16MRwa**, Bm16MR*wzm*, BmH38R*wbkF*, BmH38R*wbk*D, and BmH38R*per*) as vaccines when inoculated (10^8^ CFU/mouse) subcutaneously in mice (n = 10). In all experiments, controls were mice (n = 5 or n = 10) inoculated subcutaneously with 10^5^ CFU/mouse of Rev 1 or with 0.1 ml PBS.

### Studies in BMDM

R mutants representative of the main attenuation patterns were studied in comparison with the parental strains. BMDM were obtained from 8 to 10 week-old female C57BL/6 black mice, infected after 6 days of *in vitro* maturation (50 CFU per cell) and, at appropriate times, coverslips were processed and immunostained [71]. Briefly, R mutants were labeled using the serum of a *B. ovis* infected sheep and a Texas Red conjugated monoclonal antibody to sheep IgG and the parental strains using the serum from a *B. abortus* infected cattle and an Alexa® 594 (Molecular Probes; Eugene, OR) conjugated anti-cattle monoclonal antibody. The lysosomal-associated membrane protein 1 (LAMP1) and the endoplasmic reticulum marker calreticulin were detected using rat anti LAMP1 MoAb plus a Cys5 conjugated anti-rat IgG monoclonal antibody and a specific rabbit anti-calreticulin serum plus an Alexa® 488 (Molecular Probes) conjugated anti- rabbit IgG monoclonal antibody. The images were obtained in a confocal microscope [71]. For mutants BmH38R*wbkF*, BmH38R*wbk*D, Bm16MRwa**, and BmH38R*man*B*_core_* the images were taken at 0.5, 2, 4, 8, 12, 24, and 48 hours after infection. In addition, 24 h post-infection images were obtained for BmH38R*per*, Bm16MR*wzm*, and Bm16MR*per*.

## Supporting Information

Figure S1 Growth curves of smooth parental strains and representative *B. melitensis* R mutants in tryptic soy broth at 37Â°C. Plots labeled as A, B and C correspond to accelerated, normal or retarded growth, respectively.
(3.20 MB TIF)

Figure S2 1H-NMR spectrum of *B. abortus* 2.13 LPS polysaccharide.
(0.09 MB TIF)

Figure S3 BALB/c mice allow a better discrimination of vaccines than CD-1 mice. Plots represent the 50 (line within box), 25 and 75 (lower and upper box limits) percentiles and minimal and maximal values (lower and upper lines) of the CFU/spleen in mice vaccinated with Rev 1 or PBS and challenged with BmH38 or Bm16M.
(1.23 MB TIF)

Table S1 ORF shown not to be involved in *B. melitensis* LPS synthesis.
(0.04 MB DOC)
