# Supplementary figures and images for: Brucellosis Vaccines: Assessment of Brucella melitensis Lipopolysaccharide Rough Mutants Defective in Core and O-Polysaccharide Synthesis and Export
Source: PLoS One. 2008 Jul 23;3(7):e2760. doi: 10.1371/journal.pone.0002760 (PMC2453230; doi:10.1371/journal.pone.0002760)

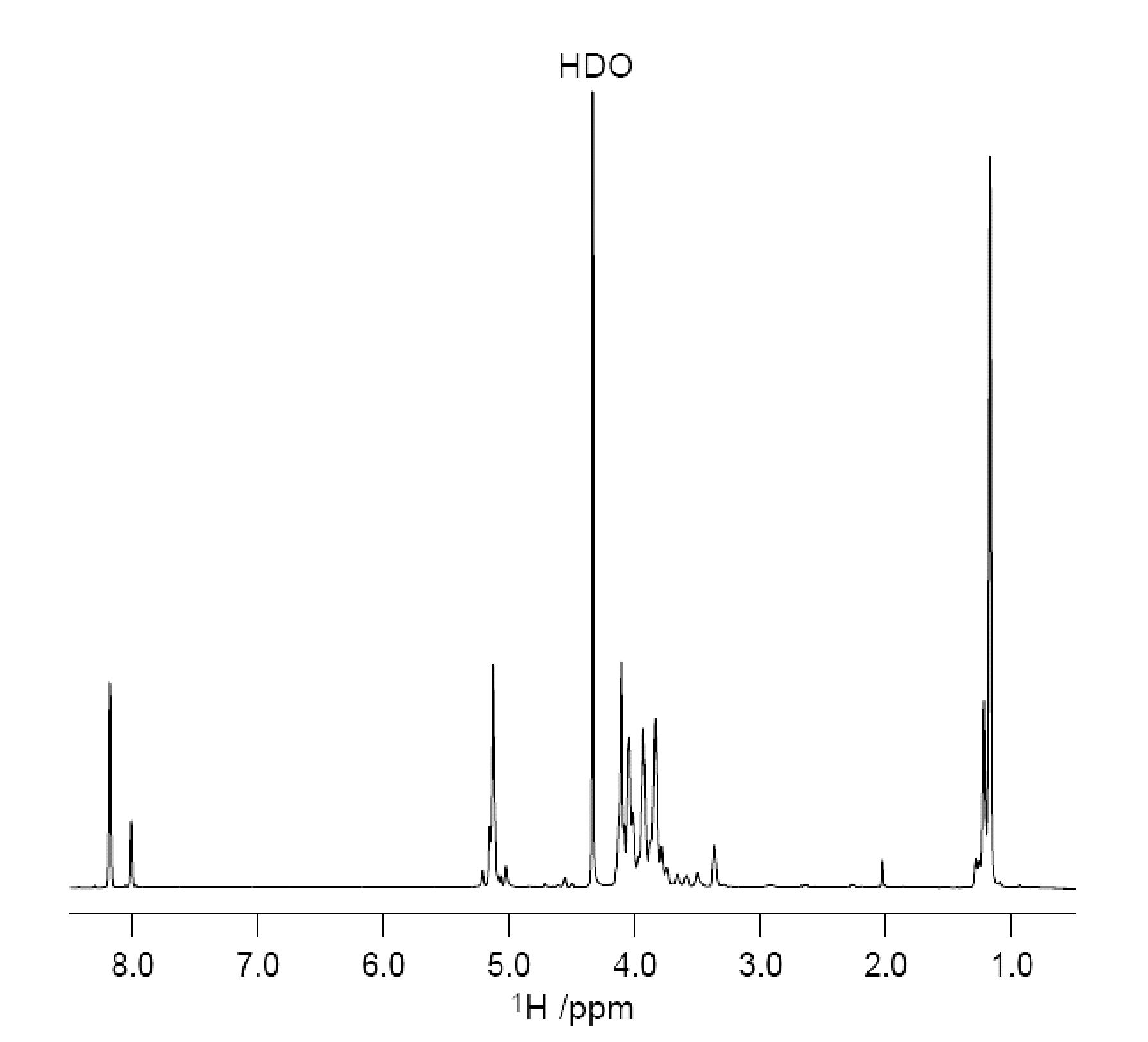

Supplement: Figure S2 — 1H-NMR spectrum of B. abortus 2.13 LPS polysaccharide. (0.09 MB TIF) [file pone.0002760.s002.tif]

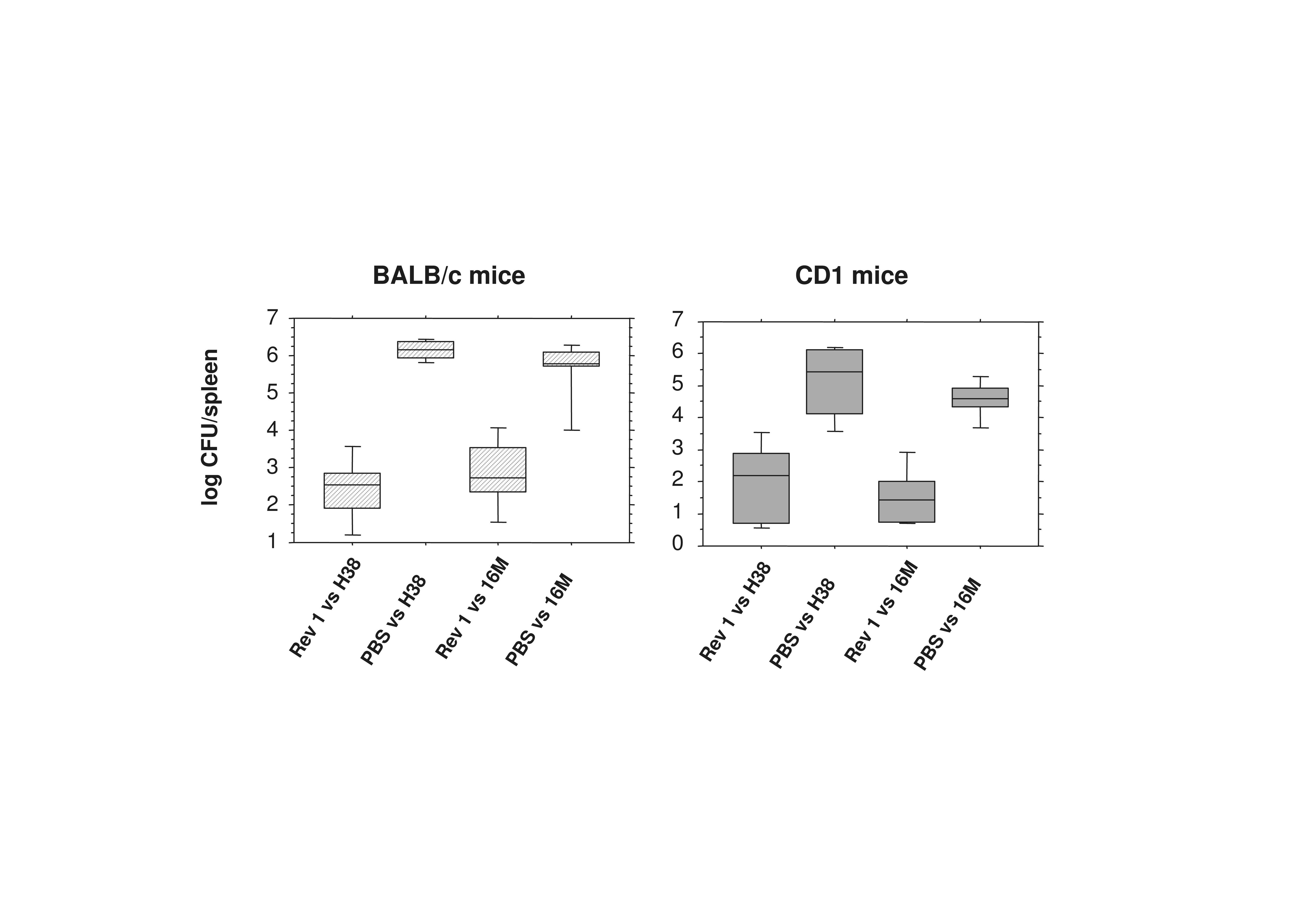

Supplement: Figure S3 — BALB/c mice allow a better discrimination of vaccines than CD-1 mice. Plots represent the 50 (line within box), 25 and 75 (lower and upper box limits) percentiles and minimal and maximal values (lower and upper lines) of the CFU/spleen in mice vaccinated with Rev 1 or PBS and challenged with BmH38 or Bm16M. (1.23 MB TIF) [file pone.0002760.s003.tif]
