# Supplementary material for: Brucellosis Vaccines: Assessment of Brucella melitensis Lipopolysaccharide Rough Mutants Defective in Core and O-Polysaccharide Synthesis and Export
Source: PLoS One. 2008 Jul 23;3(7):e2760. doi: 10.1371/journal.pone.0002760 (PMC2453230; doi:10.1371/journal.pone.0002760)
Supplement: Table S1 — ORF shown not to be involved in B. melitensis LPS synthesis. (0.04 MB DOC) [file pone.0002760.s004.doc]

**Table S1.** ORFs shown not to be involved in *B. melitensis* LPS synthesis.**^1^**

| **ORF** | **Mutagenesis** | **Annotation** |
| --- | --- | --- |
| BMEI1885 | Targeted | Transcriptional regulatory protein |
| BMEII0052 | Targeted | Sensory histidine-kinase |
| BMEII0053 | Transposon | Mg transport ATPase |
| BMEII0681 | Targeted | Virulence protein |
| BMEII0682 | Transposon | Oxacillin R associated protein |
| BMEII0684 | Targeted | Glucosamine-1-P acetyltransferase |
| BMEII0685 | Targeted | Glucosamine-fructose-6-P-aminotransferase |
| BMEII1127 | Targeted | Exopolysaccharide production protein ExoQ |
| BMEII1128 | Targeted | Succinoglycan biosynthesis protein ExoM |
| BMEII1129 | Targeted | Glycosyltransferase |
| BMEII1130 | Transposon | UDP-N-acetyl-D-mannosaminuronic transferase |
| BMEII1132 | Targeted | Succinoglycan biosynthesis transport ExoP |
| BMEII1134 | Transposon | Aminotransferase or amidase |
| BMEII1135 | Targeted | Transcriptional regulatory protein |

**^1^** All mutants derived from *B. melitensis* 16M Nal^R^
